# Editorial: Exercise and menopause: benefits, challenges and the transition to optimal management

**DOI:** 10.3389/fspor.2026.1798453

**Published:** 2026-02-26

**Authors:** Kirsty A. Roberts, David A. Low, Emma O'Donnell

**Affiliations:** 1 Research Institute for Sport and Exercise Sciences, Liverpool John Moores University, Liverpool, United Kingdom; 2 Liverpool Centre for Cardiovascular Science at University of Liverpool, Liverpool John Moores University and Liverpool Heart & Chest Hospital, Liverpool, United Kingdom; 3 School of Sport, Exercise and Health Sciences, National Centre of Sports and Exercise Medicine, Loughborough University, Loughborough, United Kingdom

**Keywords:** exercise, menopausal transistion, menopause, physical activity, quality of life, symptom management, women's health

Menopause represents a biological milestone marked by a decline in ovarian hormones, particularly oestrogen and progesterone. Rather than being a single event, menopause is best understood as a continuum of reproductive ageing. This transition begins with perimenopause, which is characterised by an increase in the irregularity of the menstrual cycle length and endocrine fluctuations, leading to the final menstrual period. Postmenopause is defined retrospectively as the period commencing 12 months after the final menstrual period (1). The timing, symptoms, duration and lived experience of the menopausal transition vary widely between individuals and across cultures, with a broad spectrum of physiological, cognitive and psychosocial changes that can profoundly affect well-being and quality of life. Menopause is not merely a reproductive event; rather, it is a complex physiological shift with systemic consequences, including vasomotor symptoms (e.g., hot flushes), sleep disturbance, mood variability, anxiety, depression, cognitive complaints (e.g., “brain fog”), metabolic and cardiovascular changes, reduced muscle mass and strength, bone loss and fatigue. As life expectancy increases, the cumulative impact of these changes becomes increasingly salient, with women worldwide often spending up to one-third of their lives in postmenopause. This underscores the importance of interventions that promote healthy ageing.

Despite its significance, menopause remains under-recognised and under-addressed globally, particularly in low- and middle-income countries, where cultural taboos, limited access to healthcare and systemic neglect hinder open discussion and timely care. Exercise is increasingly recognised not only as a lifestyle recommendation but as an accessible, multidimensional therapeutic strategy that is capable of addressing many of the challenges associated with midlife, including cardiovascular risk, changes in body composition, mood disturbances, sleep quality and cognitive function. The contributions to this Special Issue, *Exercise and Menopause: Benefits, Challenges and the Transition to Optimal Management*, provide compelling evidence and practical insights into how exercise can support women throughout the menopausal transition.

Global and cultural perspectives emphasise that menopause is not experienced in isolation. In *Breaking the Silence and Building Strength*, Delanerolle et al. highlighted how religious beliefs, social norms and policy gaps can limit women's engagement in physical activity across Asia, Africa and the Middle East. The authors also identified community-based programmes, corporate initiatives and digital platforms as promising, culturally adaptable approaches, demonstrating that exercise can be both a cost-effective and socially sensitive strategy for managing vasomotor, cognitive and psychosocial symptoms. Complementing this perspective, Snani et al. examined menopausal health among Tunisian women, reporting that fatigue and psychosocial symptoms are prevalent across the menopausal transition, while physical activity and healthier body composition are consistently associated with better quality of life. Notably, the protective effects of exercise were shown to be strongest in premenopausal women, suggesting that engagement in exercise may help to mitigate menopausal challenges.

Exercise for musculoskeletal and metabolic health also emerges as a central theme across this research topic. The menopausal transition is associated with accelerated loss of skeletal muscle mass, bone demineralisation and increased cardiometabolic risk, highlighting the need for exercise strategies that address multiple physiological systems simultaneously. Delanerolle et al. proposed that innovative exercise modalities, such as non-contact boxing, may offer advantages over traditional exercise approaches by integrating resistance, impact, coordination and aerobic components within a single modality. These approaches may therefore support the preservation of lean mass, the maintenance of bone density, improvements in balance and enhancements in psychological well-being in an efficient and engaging format.

Similarly, Wochna et al. reported that Nordic walking using resistance shock absorber poles can beneficially influence muscle stiffness and elasticity, particularly in the upper limbs, thereby supporting mobility and functional independence in postmenopausal women. At a broader population level, a large network meta-analysis conducted by Zhang et al. demonstrated that aerobic, resistance, combined and mind-body exercise modalities confer distinct benefits across metabolic syndrome risk factors. These findings reinforce the importance of tailoring exercise prescriptions to individual health priorities and risk profiles, rather than adopting a one-size-fits-all approach.

At the mechanistic end of the research spectrum, Dai et al. described a novel 12-week intervention protocol combining repetitive transcranial magnetic stimulation with low-intensity blood flow restriction resistance training to investigate central and peripheral adaptations related to skeletal muscle mass and physical function in community-dwelling postmenopausal women. These mechanistic investigations are critical for advancing our understanding of the pathways through which exercise interventions can preserve muscle health and physical function during menopause and for informing the development of targeted, evidence-based exercise strategies.

The cognitive and psychosocial benefits of exercise also emerge as an important theme in this research topic. Jóźwiak et al. reported that resistance and endurance training are associated with improvements across multiple cognitive domains, whereas combining exercise with time-restricted eating does not confer additional cognitive advantages. Consistent with these findings, Kuck and Hogervorst demonstrated that engagement in physical activity and yoga is independently associated with lower perceived stress and a better psychosocial quality of life, while menopausal hormone therapy alone is not associated with improved psychosocial outcomes. Collectively, these studies support exercise as a central lifestyle strategy for cognitive and mental health during postmenopause, particularly when adapted to individual needs, preferences and contexts.

Mechanistic insights into vasomotor symptoms, particularly hot flushes, a hallmark feature of the menopausal transition, remain comparatively under-investigated. To address this gap, Roberts et al. presented a protocol for a mechanistic study comparing the function and structure of skin blood vessels and sweat glands in pre- and postmenopausal women and in postmenopausal women with and without hot flushes. Understanding these peripheral vascular and sudomotor differences may help clarify the physiological mechanisms underpinning hot flushes and inform the development of more targeted interventions, including both lifestyle- and pharmacologically-based approaches.

Across these contributions, several clear and interlinked themes emerge. First, exercise is inherently multidimensional, offering concurrent benefits for physical, metabolic, cognitive and psychosocial health during the menopausal transition. Second, personalisation appears central to effectiveness: exercise modality and overall programme design should be tailored to the individual's health status, goals and contextual factors, rather than being applied as a uniform prescription. Third, innovation and engagement matter. Approaches such as non-contact boxing, Nordic walking and low-intensity blood flow restriction resistance training illustrate how creative and contextually relevant exercise interventions can enhance both adherence and physiological impact. Finally, equity and cultural sensitivity are essential to ensure that exercise-based strategies are accessible, acceptable and effective for women in diverse global settings, particularly where systemic and sociocultural barriers persist.

Looking ahead, future research should continue to refine stage-specific exercise prescriptions, thoughtfully integrate multimodal interventions and prioritise scalable community- and digitally-delivered programmes that broaden access. In parallel, policymakers, healthcare systems and clinicians must recognise exercise not as an optional lifestyle adjunct but as a foundational component of midlife women's health, embedded within standard care pathways alongside pharmacological and non-pharmacological strategies.

In conclusion, the studies presented in this Special Issue collectively reinforce exercise as a robust, evidence-based, and adaptable intervention to support women throughout the menopausal transition. By integrating mechanistic insight, innovative exercise modalities and global perspectives, this body of work advances the field towards more personalised, equitable and sustainable approaches to optimising health and well-being in midlife and beyond.
